# Bioinspired hierarchical helical nanocomposite macrofibers based on bacterial cellulose nanofibers

**DOI:** 10.1093/nsr/nwz077

**Published:** 2019-06-21

**Authors:** Huai-Ling Gao, Ran Zhao, Chen Cui, Yin-Bo Zhu, Si-Ming Chen, Zhao Pan, Yu-Feng Meng, Shao-Meng Wen, Chuang Liu, Heng-An Wu, Shu-Hong Yu

**Affiliations:** 1 Division of Nanomaterials & Chemistry, Hefei National Laboratory for Physical Sciences at the Microscale, CAS Center for Excellence in Nanoscience, Hefei Science Center of CAS, Department of Chemistry, University of Science and Technology of China, Hefei 230026, China; 2 CAS Key Laboratory of Mechanical Behavior and Design of Materials, Department of Modern Mechanics, CAS Center for Excellence in Complex System Mechanics, University of Science and Technology of China, Hefei 230027, China

**Keywords:** bioinspired, nanocomposite, hierarchical helical macrofibers, strength and toughness, bacterial cellulose

## Abstract

Bio-sourced nanocellulosic materials are promising candidates for spinning high-performance sustainable macrofibers for advanced applications. Various strategies have been pursued to gain nanocellulose-based macrofibers with improved strength. However, nearly all of them have been achieved at the expense of their elongation and toughness. Inspired by the widely existed hierarchical helical and nanocomposite structural features in biosynthesized fibers exhibiting exceptional combinations of strength and toughness, we report a design strategy to make nanocellulose-based macrofibers with similar characteristics. By combining a facile wet-spinning process with a subsequent multiple wet-twisting procedure, we successfully obtain biomimetic hierarchical helical nanocomposite macrofibers based on bacterial cellulose nanofibers, realizing impressive improvement in their tensile strength, elongation and toughness simultaneously. The achievement certifies the validity of the bioinspired hierarchical helical and nanocomposite structural design proposed here. This bioinspired design strategy provides a potential platform for further optimizing or creating many more strong and tough nanocomposite fiber materials for diverse applications.

## INTRODUCTION

High-performance biomass-based nanocomposites are emerging as advanced renewable and sustainable materials for future structural and functional applications [1–9]. Bio-sourced nanocellulosic materials, the most abundant raw material systems on earth, have attracted tremendous scientific and commercial attention recently due to their attractive combination of many inherent merits in terms of biodegradability, low density, thermal stability, global availability from renewable resources, as well as impressive mechanical properties [10,11]. These features make them promising candidates for the development of mechanically robust, sustainable and biocompatible materials for diverse applications [12–14].

Nanofibrillated cellulose (NFC) [13] and cellulose nanocrystals (CNC) [15] obtained from plants and bacterial cellulose (BC) nanofibers obtained via bacterial fermentation [16] represent a remarkable class of nature-derived nanofibers with superior intrinsic mechanical properties owing to their high degree of polymerization and crystallinity. These extremely fine natural polymeric nanofibers have been intensively investigated for fabricating high-performance macrofibers. Various strategies, such as flow-assisted assembly [13], combining wet-spinning with mechanical stretching [17,18] or chemical crosslinking [14], mixing synergetic constituents together [19], have been pursued to strengthen the nanofiber alignment or enhance the interfibrillar interactions, etc. As a result, significant enhancements in strength and stiffness have been achieved in the resultant nanocellulose-based macrofibers. However, as strength and toughness are always mutually exclusive for manmadex structural materials [20], almost all the achievements ultimately came at the expense of elongation and toughness of the obtained macrofibers. For example, mechanical stretching can improve the NFC/CNC orientation resulting in marked improvement of tensile strength and stiffness, but meanwhile leads to obvious embrittlement and low failure strain [21,22]. Generally, compared with strength and stiffness, elongation and toughness are even more critical for fiber materials, especially for those relative to textile applications [23–25]. Therefore, this dilemma is quite common for previously reported nanocellulose-based macrofibers. Besides pursuing high tensile strength, further improving their elongation and toughness is still a significant challenge.

Nature can always provide inspirations for us to remedy this troublesome conflict between strength and toughness [26]. The widespread biosynthesized fibers, ranging from various lignocellulosic fibers in plants [11] to spider silk [27], collagen fibers and animal hairs [28], are all characterized by exceptional combinations of high tensile strength and toughness. This performance combination is mainly attributed to their hierarchical helical structures across multiple length scales with stiff and strong nanoscale fibrous building blocks embedded in softer and energy-dissipating matrices [29]. These soft matrices are supposed to play critical roles in the mechanical behavior by providing favorable interfaces between the fibrous units. Typically, NFCs in the outer cell wall layer (S2 layer) of plants are aligned and embedded in a matrix of lignin and hemicellulose to form strong and tough nanocomposite helical fibers.

**Figure 1.**
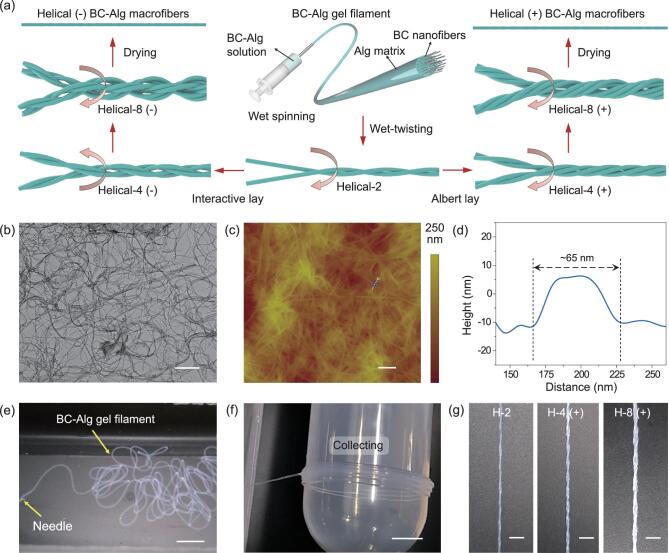
Fabriction process of the hierarchical helical BC–Alg macrofibers. (a) Schematic illustration of the fabrication process of the bioinspired hierarchical helical BC–Alg macrofibers. At each hierarchical level, every two sub-level gel filaments are twisted together to prepare a higher-level helical fiber. Helical-2, 4 and 8 indicate helical fibers composed of 2, 4 and 8 original filaments, respectively. The helical fibers with the same twist direction (Albert lay) at each level are defined as helical (+). The helical fibers with opposite twist directions (interactive lay) at each level are defined as helical (−). (b) Transmission electron microscope (TEM) image of the dispersed BC nanofibers. Scale bar, 500 nm. (c, d) Atomic force microscopy (AFM) image and its corresponding height profile (dotted line) of the dispersed BC nanofibers. The measured diameter of a typical BC nanofiber is about 65 nm. Scale bar in (c), 500 nm. (e) Photograph shows that a continuous BC–Alg gel filament is extruded through a capillary needle into a coagulation bath of CaCl_2_ aqueous solution. Scale bar, 10 mm. (f) Photograph shows that a continuous BC–Alg gel filament is collected by a winding roller. Scale bar, 10 mm. (g) Photographs show the twisted state of a bundle of BC–Alg gel filaments after wet-twisting processes at each hierarchical level. Scale bars, 5 mm.

Herein, inspired by the above motivations, we propose a hierarchical helical and nanocomposite structural design strategy to fabricate mechanically robust macrofibers. The obtained macrofibers are expected to be composed of helically aligned BC nanofibers embedded in a soft biopolymer matrix (Fig. 1a). By combining a facile wet-spinning process with a subsequent multiple wet-twisting and drying procedure, the structural and mechanical features in natural biosynthesized fibers are supposed to be transferred into the designed artificial macrofibers, achieving hierarchical helical nanocomposite macrofibers with a simultaneous improvement of tensile strength, elongation and toughness, which is challenging for most previously reported nanocellulose-based macrofibers. The expected combination of desirable mechanical properties will endow the macrofibers with promising potential for further advanced applications.

## RESULTS AND DISCUSSION

### Fabrication and structural characterization

As an alternative to plant-derived NFC and CNC, BC nanofibers are much purer, have larger aspect ratios and higher degrees of crystallinity and polymerization [30–32], which make them a particularly appealing kind of building block for fabricating mechanically robust macrofibers. Here, dispersed BC nanofibers with diameters of approximately 60 nm and lengths of dozens of micron (Fig. 1b–d and Supplementary Fig. 1a) were utilized as the structural units to prepare our hierarchical helical nanocomposite macrofibers. Sodium alginate (Alg), a biodegradable and biocompatible anionic polysaccharide with abundant carboxylic acid groups, was chosen as the soft matrix between BC nanofibers. The hydroxyl groups in BC [31] can interact with the carboxylic acid groups of Alg, forming strong hydrogen bonds (Supplementary Fig. 1b). Thus, good interfacial interaction between BC nanofibers and Alg can be achieved. This interfacial interaction is supposed to be beneficial for stress transfer between the two components, which is essential for good mechanical properties of the relevant composites [33].

For fabricating the hierarchical helical nanocomposite macrofibers, aqueous dispersions of BC–Alg mixture were first spun into continuous BC–Alg gel filaments consisting of uniaxial orientation of BC nanofibers embedded in the Alg matrix (Supplementary Fig. 1c). A facile wet-spinning method widely used in previous work to facilitate uniaxial orientation of other nanofibers under shear force from flows [34] was applied here. Unlike the classical wet-spinning method applied in industry, where high-concentration polymer solutions are extruded through thousands of capillaries into coagulation baths to produce yarns of fibers in huge amounts, the wet-spinning process used here was simplified to demonstrate our study. To be specific, BC–Alg dispersions were extruded through a single capillary needle into coagulation baths of CaCl_2_ aqueous solution to form continuous BC–Alg gel filaments (Fig. 1e and Supplementary Movie 1). During the extrusion process, BC nanofibers were forced to align parallel to the long axis of the needle under local shear force at the solid boundaries of the nozzle [17]. Then the orientation of BC nanofibers was fixed immediately upon Ca^2+^-induced coagulation of the Alg matrix (Supplementary Fig. 1d). In order to introduce hierarchical helical structure into the ultimate macrofibers, the obtained BC–Alg gel filaments (Fig. 3f) were then twisted together according to a proposed multilevel wet-twisting process as illustrated in Fig. 1a. For simplicity, every two sub-level gel filaments were twisted together at each hierarchical level (Fig. 3g, Supplementary Fig. 2 and Supplementary Movie 1) to verify our design strategy. The twisted gel filaments with different hierarchical levels were then air-dried to obtain hierarchical helical nanocomposite macrofibers with dense structures.

**Figure 2.**
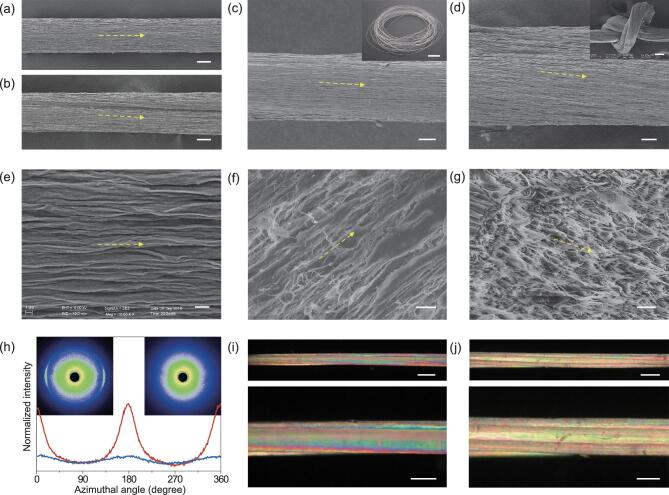
Structural characterization of the hierarchical helical BC–Alg macrofibers. (a-d) Lateral surface SEM images of single (a), helical-2 (b), helical-4 (+) (c) and helical-8 (+) (d) BC–Alg macrofibers, where a slightly twisted surface texture (indicated by the yellow arrows) can be seen for the helical BC–Alg fibers. Scale bars, 20 μm. The inset photograph in (c) is a roll of helical-4 (+) BC–Alg macrofiber. Scale bar, 5 mm. The inset SEM image in (d) shows a knotted helical-8 (+) BC–Alg macrofiber. Scale bar, 100 μm. (e-g) High-magnification lateral surface (e), longitudinal-sectional (f) and cross-sectional SEM images (g) of a typical single BC–Alg filament, showing the parallelly oriented BC nanofibers (indicated by the yellow arrows) along the long axis of the filament. Scale bars, 2 μm. (h) Azimuthal intensity profiles of the (200) scattering plane of the wide-angle X-ray scattering diffractograms of a bundle of helical BC–Alg macrofibers (right inset) and a BC–Alg film with randomly oriented BC nanofibers. (i, j) Optical micrographs with different magnifications of a helical-4 (+) macrofiber (i) and a helical-8 (+) macrofiber (j) between crossed polarizers, which both reveal typical birefringence with slightly twisted morphology. Scale bars, 200 μm for the upper and 100 μm for the lower micrographs, respectively.

Scanning electron microscopy (SEM) observation revealed that both the Albert lay (+) and interactive lay (−) helical BC–Alg macrofibers after drying presented a slightly twisted alignment of surface textures (Fig. 2a–d and Supplementary Figs 3–5). The interface between the original separate filaments was ambiguous with only a few packing defects or voids, which should disappear during the drying process. Before drying, the gel filaments were actually hydrogels, containing large amounts of water. The calcium alginate macromolecular chains in the gel filaments were in a relatively relaxed state. The gel filaments had a good wettability due to their high water content. When several single gel filaments were twisted together, a thin water film on the surface would further bind the gel filaments closer to each other due to surface tension. In this state, the outermost polymer segments would penetrate into other surfaces as a result of their high local mobility [35]. Thus, during the drying process, the entanglement (interpenetration) of the surface segments was supposed to happen under the van der Waals force among them. Therefore, the original interfaces among the gel filaments disappeared slowly, and the original separate gel fibers integrated into relatively dense single macrofibers with only a few voids. Note that the helical macrofibers exhibit good flexibility and knitting property (Fig. 2d inset), indicating their promising potential for textile applications. From a close-up of the surface of a single BC–Alg filament shown in Fig. 2e, a crumpled texture with the direction along its long axis was observed, which was attributed to lateral contraction during the drying process, because both ends of the macrofibers were fixed before

drying. This lateral contraction should further contribute to the orientation of the embedded BC nanofibers. As we expected, BC nanofibers in the single BC–Alg filament were homogeneously distributed and oriented almost parallel along its long axis (Fig. 2f, g and Supplementary Fig. 6a–c). A high-magnification cross-sectional SEM image shows that individual BC nanofibers have been pulled out from the Alg matrix on the fracture surface (Fig. 2g), indicating efficient stress transfer and great energy dissipation during rupture.

Identification of the orientation of BC nanofibers in the microfibers was further assessed via wide-angle X-ray diffraction (WAXD). In stark contrast to that of the BC–Alg film with randomly oriented BC nanofibers in the horizontal direction (Supplementary Fig. 6d), the (200) reflection used to quantify the orientation of cellulose crystals [13] was presented as an arc pattern in the diffractogram of the dried BC–Alg macrofibers and the azimuthal intensity profiles of the (200) reflection were prominent (Fig. 2h), suggesting the highly aligned orientation of BC nanofibers in the nanocomposite BC–Alg macrofibers [36]. Moreover, the strong orientation of BC nanofibers in the dried hierarchical helical macrofibers was then confirmed by the brilliant color under cross-polarized light due to the birefringence phenomenon of BC nanofibers caused by their oriented structure [17]. As shown in Fig. 2i, j and Supplementary Figs 7–9, both the helical-4 and helical-8 macrofibers with Albert lay (+) and interactive lay (−) reveal obvious brilliant colors with slightly twisted directions. Note that each original BC–Alg filament with a twist state in the macrofibers can be distinguished. These results can partly confirm the helical spatial distribution of BC nanofibers in nanocomposite macrofibers over large-scale dimensions.

### Mechanical properties

**Figure 3.**
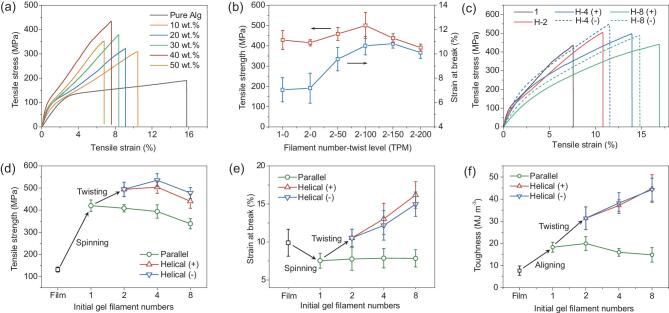
Mechanical properties of the hierarchical helical BC–Alg macrofibers. (a) The typical stress–strain curves of the dried single BC–Alg filaments composed of different contents of BC nanofibers. (b) The tensile strength as a function of twist level of the helical-2 fibers. (c) The typical stress–strain curves of the hierarchical helical BC–Alg macrofibers with different hierarchical structures. (d-f) The tensile strength (d), strain at break (e) and toughness (f) as functions of hierarchical levels of the BC–Alg macrofibers. All the error bars represent the s.d. of at least six replicate measurements.

Systematic mechanical investigations were carried out to establish a quantitative correlation between the mechanical performance and the designed structures. It was found that the tensile strength of

the dried BC–Alg filaments showed a consistent increase with increasing BC nanofiber content up to a weight ratio of 40 wt.% and then rapidly dropped (Fig. 3a and Supplementary Fig. 10a). The optimal filament showed more than double and nearly four times the original tensile strength of the neat Alg filament and the disordered BC–Alg film with the same BC content, respectively (Supplementary Fig. 10). These results clearly certify the contribution of BC alignment to the ultimate mechanical properties, and also reveal that a proper amount of soft matrix is crucial for efficient stress transfer between BC nanofibers, leading to the impressive mechanical properties of the ultimate nanocomposite filaments (Fig. 3b). The optimized twist level was further identified to be 100 twists per meter (TPM) for the helical-2 BC–Alg macrofibers in consideration of the optimum combination of strength and elongation. As shown in Fig. 3b, both the strength and strain at break rise similarly to the rising twist levels before a critical point (2–100) and then begin to decrease. It can be hypothesized that increasing the twist level would cant the BC nanofibers and improve the inter-filament interaction under axial tension leading to an increase in the macrofiber strength before the optimum point, after which the nanofiber obliquity in the macrofiber becomes unsuitable, leading to a drop in the macrofiber strength. This phenomenon matches the mechanical behavior found in traditional twisted yarns [37]. Besides, the pre-stressing force induced by wet-twisting and drying process might also correlate to the strength of the resultant macrofibers.

The effect of helical hierarchy on the mechanical properties of the resultant macrofibers was further investigated. We found that the Albert lay (−) BC–Alg macrofibers exhibited slightly higher tensile strength and smaller elongation, respectively, than those of the interactive lay (+) BC–Alg macrofibers at each hierarchical level (Fig. 3c–e and Table 1). Note that the tensile strength continuously increased for the helical-4 macrofibers, then dropped for the helical-8 macrofibers, while both Albert lay (+) and interactive lay (−) BC–Alg macrofibers showed continuous increase of strain at break as the hierarchical levels increased (Fig. 3c–e and Table 1). Consequently, impressive enhancement of toughness compared with those of the single BC–Alg filament and the disordered BC–Alg film was achieved, respectively (Fig. 3f and Table 1). In sharp contrast, all the macrofibers made from the same number of gel filaments without twisting show much smaller tensile strength, strain at break (nearly no change) and toughness than those of the helical macrofibers (Fig. 3d–f and Table 1). It is worth highlighting that in most traditional nanocomposites, higher strength is typically achieved at the expense of strain at break and toughness. However, we achieved a simultaneous increase of strength, elongation and toughness in our bioinspired macrofibers. This mechanical enhancement effect was also achievable for the bioinspired macrofibers conditioned at different relative humidity (Supplementary Fig. 11 and Supplementary Table 1). These experimental findings provide direct evidence for the positive contribution of our bioinspired hierarchical helical and nanocomposite structural design to the mechanical properties of resultant macrofibers. The strength decreases of the helical-8 and untwisted macrofibers are supposed to be mainly caused by some inevitable packing voids or defects derived during their drying processes. In these processes, the wet gel filaments changed into dried macrofibers with a large radial shrinkage (about 10 times) due to the loss of plenty of water. Thus, some packing voids or defects among the gel filaments are considered to be inevitable during this process, which would have a negative impact on the mechanical strength of both the helical fibers and the untwisted macrofibers. The increases of tensile strength of the helical-2 and helical-4 fibers should be attributed to the relatively larger contribution of the bioinspired structural design, and this negative effect becomes dominant with the increase of initial gel filament numbers.

**Table 1.** Comparation of mechanical properties of the fabricated BC–Alg macrofibers with different structural features. All the values were the mean value of at least six replicate measurements.

Initial gel filament numbers		1	2	4	8
**Strength (MPa)**	Helical (+)	420.4	494.0	504.2	440.3
	Helical (−)			535.4	477.6
	Parallel		408.9	394.2	338.7
**Strain (%)**	Helical (+)	7.5	10.5	13.0	16.2
	Helical (−)			12.2	15.0
	Parallel		7.8	7.9	7.9
**Toughness (MJ m^−^^3^)**	Helical (+)	18.3	31.4	37.1	44.8
	Helical (−)			38.3	44.3
	Parallel		20.0	16.0	14.8

**Figure 4.**
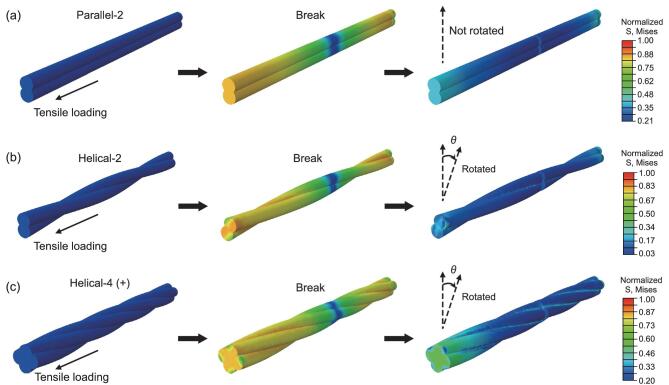
Mechanical simulations of the fiber models with different structural features under tension loading. (a-c) Non-linear finite element simulations display three kinds of fiber models under uniaxial tension processes. Note that both the helical-2 and helical-4 (+) fiber models would rotate in the tension processes before break. *θ* indicates the rotation degree of the simulated helical fibers from initial tension to rupture.

Owing to the helical structure, both the Albert lay (+) and interactive lay (−) BC–Alg macrofibers would rotate under tensile loading, and the Albert lay (+) case is especially prone to this tendency according to the traditional theory for rope and rope-like materials [38]. During this rotation process, each helically distributed filaments in the macrofibers is gradually straightened before break, leading to elongation increase of the whole helical BC–Alg fibers. It is assumed that helical fibers with higher hierarchical levels exhibit larger elongation potential due to the multilevel rotational deformations. Non-linear finite element simulations further revealed a good consistency with this speculation. In sharp contrast to the untwisted fiber model, both the helical-2 and helical-4 fiber models revealed an obvious rotation phenomenon under uniaxial tension (Fig. 4, Supplementary Fig. 10 and Supplementary Movie 2), resulting in conspicuous improvement of tensile deformation before the fibers break relative to the untwisted one (Supplementary Fig. 10c). Thus, we can infer why the elongation of the interactive lay (−) BC–Alg macrofibers was found to be a little smaller relative to the Albert lay (+) BC–Alg macrofibers and much larger than that of the untwisted macrofibers. Moreover, during uniaxial tension, multiscale deformation with interfacial frictional sliding of the discontinued BC nanofibers in the Alg matrix at each helical hierarchy would happen synchronously, giving rise to much energy dissipation [25,39,40]. Consequently, attractive improvements of toughness for both the Albert lay (+) and interactive lay (−) macrofibers were achieved (Fig. 3e, f).

**Figure 5.**
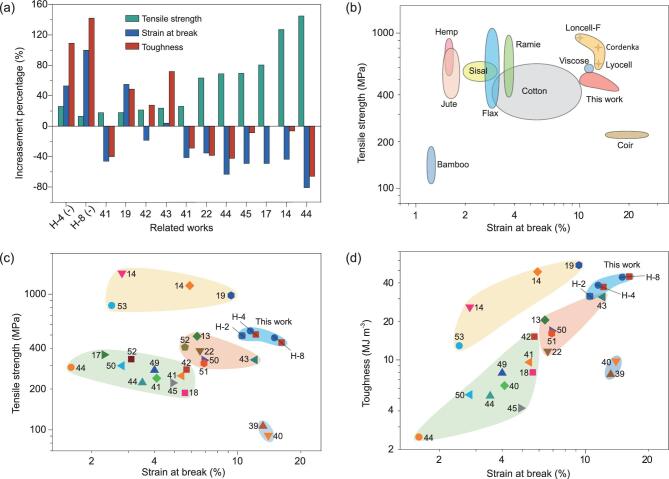
Comparison of the mechanical properties of our designed hierarchical helical macrofibers with their counterparts. (a) Comparison of mechanical enhancement of the tensile strength, strain at break and toughness of our hierarchical helical macrofibers and previously reported cellulose-based macrofibers [14,17,19,22,41–45]. (b) Ashby diagram of the tensile strength vs. strain at break of our hierarchical helical fibers compared with several mature industrial regenerated cellulose fibers and a wide range of biosynthetic cellulose-based fibers [46–48]. (c, d) Ashby diagram of specific strength vs. strain at break (c) and toughness vs. strain at break (d) of our hierarchical helical fibers and previously reported cellulose-based fibers [13,14,17–19,22,39–45,49–53]. Numbers in the charts stand for relevant references. Data plotted in the charts (c, d) were extracted from the maximum values given or estimated from stress–strain curves in these references. The increasement percentages of the tensile strength, strain at break and toughness in (a) were calculated by using the difference between the given values of the fibers without and with relevant design strategies.

It is obvious that the obtained hierarchical helical macrofibers exhibited a distinguishing mechanical improvement with a simultaneous increase of strength, elongation and toughness relative to the untwisted macrofibers, providing a valuable solution for the traditional dilemma of most manmade fiber materials. It can be found that in most previously reported nanocellulose-based macrofibers, there tends to be a regular phenomenon that increasing the tensile strength is always achieved at the expense of elongation and toughness. In contrast, the elongation and toughness increased by more than ∼50% and 100%, respectively, for the helical-4 (−) macrofibers with simultaneous increase of tensile strength (Fig. 5a). The unique property combination of our designed macrofibers should be attributed to the synergistic effects of the bioinspired hierarchical helical and nanocomposite structure. As shown in Fig. 5b, c and Supplementary Table 2, though the achieved maximum tensile strength (∼535 MPa) is still lower than those of some mature industrial regenerated cellulose fibers (Lyocell, Cordenka and Ioncell-F fibers) and several nanocellulose-based spun fibers [14,19,53], it is comparable to those of high-quality biosynthesized fibers and outperforms those of most nanocellulose-based macrofibers in previous work. Furthermore, the elongation (with a maximum average value of ∼16%) surpasses nearly all those of the biosynthesized and the reported manmade nanocellulose-based fibers. As a result, the bioinspired hierarchical helical macrofibers display impressive

mechanical superiority, especially when considering both the toughness (with a maximum value of ∼45 MJ m^−3^) and elongation (Fig. 5d and Supplementary Table 2), certifying the validity of the bioinspired hierarchical helical and nanocomposite structural design proposed here. Additionally, it should be noted that though the designed bioinspired hierarchical helical macrofibers share some similarities in terms of helical hierarchy and performance characteristics with traditional rope materials [38], the benefits derived from the intrinsic merits of nanoscale building blocks and the superior nanocomposite structure are prominent.

## CONCLUSION

In this study, with regard to the general problem of low elongation or brittleness of previously reported nanocellulose-based macrofibers, we reported a bioinspired hierarchical helical and nanocomposite structural design strategy to fabricate mechanically robust macrofibers by combining a facile wet-spinning process with a subsequent multiple wet-twisting. The resultant macrofibers exhibit a hierarchical helical structure with good alignment of BC nanofibers embedded in a soft Alg matrix in the macrofibers. This structural feature is recognized to give rise to the distinguishing mechanical improvement of the resultant nanocomposite macrofibers. The bioinspired structural design strategy presented here is simple, eco-friendly and valid, representing a promising platform for the development of high-performance nanocomposite fiber materials for future structural or functional applications, such as advanced textiles.

## METHODS

### Fabrication of the hierarchical helical BC–Alg macrofibers

Dispersed BC nanofibers were purchased from Qihong Technology Co., Ltd (Guilin, Guangxi,

China). SA were purchased from Aladdin Chemical Reagent Co. and used without further purification. Calcium chloride (CaCl_2_) was purchased from Sinopharm Chemical Reagent Co. Alg solution (20 mg ml^−1^) was mixed with BC nanofiber dispersions (4.5 mg ml^−1^) by intense stirring for ∼60 min at room temperature to prepare the BC–Alg dispersions with certain contents of BC (0 to 50 wt.%), followed by vacuum-pumping treatment to remove air bubbles. The resulting BC–Alg dispersions were then loaded into a plastic syringe and extruded through a capillary needle (a steel tube with an inner diameter of 0.41 mm and a length of 15 cm) into a CaCl_2_ coagulation bath (0.1 M) to form continuous BC–Alg gel filaments. The injection was controlled by an air pump operating at a pressure of ∼20 psi. The continuous spun gel filaments were rolled onto a drum (Fig. 1e, f and Supplementary Movie 1) after soaking in the coagulation bath for 10 min. The collected gel filaments were then immersed in deionized water (DIW) to remove excess Ca^2+^. Afterwards, two single BC–Alg gel filaments were hung on the rotator of a homemade twisting machine with two ends fixed without any drawing (Supplementary Fig. 2), and then twisted together into hierarchical helical BC–Alg macrofibers with certain twist levels according a multilevel wet-twisting process (Fig. 1a, g and Supplementary Movie 1). The optimized twist levels for helical-2, 4 and 8 macrofibers are 100, 67 and 45 TPM, respectively, to achieve similar twist angles for them. At each hierarchical level, every two sub-level filaments were twisted together. The rotation directions were changed to obtain helical (+)/(−) fibers. The obtained gel macrofibers were finally hung on a shelf at room temperature with a relative humidity of 50% for air drying. In this process, we first fixed one end of the gel macrofibers on a shelf and let the fibers suspend by gravity, then we fixed the other end without any drawing. The untwisted macrofibers consisting of different numbers of single filaments were prepared by binding these single filaments together in parallel and drying them under the same conditions as the hierarchical macrofibers. The disordered BC–Alg film was prepared by self-evaporation of BC–Alg dispersions containing 40 wt.% BC nanofibers. The dried film was then immersed into a CaCl_2_ solution (0.1 M) for one hour followed by washing and air drying.

### Structure characterizations

TEM with a Hitachi H-7650 apparatus at an acceleration voltage of 120 kV was used to observe the BC nanofibers. The microstructure of the BC nanofibers, the obtained BC–Alg macrofibers and films were observed by SEM (Zeiss Supra 40) at an acceleration voltage of 5 kV. AFM measurements were carried out on a Veeco DI Nanoscope MultiMode V system in tapping mode. X-ray diffraction (XRD) patterns were achieved on a PW1710 instrument with CuKα radiation (*λ* = 0.154 06 nm). Fourier transform infrared spectroscopy (FTIR) spectra were obtained from a Bruker Vector-22 FTIR spectrometer at room temperature.

### Orientation characterizations

An optical microscope image between crossed polarizers was obtained with a polarizing microscope (Leica DM2700P, Germany) equipped with a Leica MC190 HD camera. 2D WAXD measurements were carried out to monitor the evolution of structures. The X-ray wavelength was 0.154 nm and a Mar345 CCD detector (150 × 150 pixels) was employed to collect the time-resolved 2D WAXD patterns. A bundle of BC–Alg macrofibers were placed in a sample holder perpendicular to the X-ray beam. The distance between the detector (Mar 345) and the sample was 195.00 mm. A typical acquisition time was 60 s. The patterns were corrected for air scattering and background. Fit2D software from the European Synchrotron Radiation Facility was used to analyze the data.

### Mechanical testing

For samples tested at different relative humidity (RH), the samples were first conditioned at 45% RH, 50% RH and 65% RH, respectively, for at least 24 h prior to testing. Then the samples were tested immediately in uniaxial tension at room temperature using an Instron 5565 A equipped with 10 N and 500 N load cells. For the macrofibers dried in a completely dry environment, they were conditioned in an 80°C oven for 24 h first, and then roasted using an infrared lamp beside the samples when they were under mechanical testing to avoid absorbing moisture from the air. At least six specimens were tested for all the values presented. The specimens were cut into ∼30 mm long pieces. Tests were performed at a loading rate of 1 mm min^−1^ with a gauge length of ∼10 mm. The tensile strength was calculated by using the fiber cross-sectional areas from optical microscope images (cross-checked with SEM images) that were measured by IMAGE J.

### Finite element analysis

Three models with different structures consisting of two parallel cylinders, two spiral cylinders and two secondary spiral cylinders, respectively, were built as shown in Fig. 3g–i and Supplementary Fig. 12a. 3D non-linear finite element simulations were performed using the commercial software ABAQUS. In the simulation, cohesive zone models were used for half of the fiber structure to model the failure of our designed material structures, where a bi-linear traction–separation (TS) law was adopted, as shown in Supplementary Fig. 12b. The fibers with isotropic elastic modulus *E* = 14.5 GPa, Poisson ratio *ν* = 0.31 suffered from elastic and plastic deformation before failure. The parameters *K_nn_*, *K_ss_*, and *K_tt_* were set to be 14.5 GPa, which represent the interfacial stiffness relating the nominal (*n*), first (*s*) and second (*t*) shear directions to the displacement, respectively. A mixed-mode fracture was taken into consideration due to the fiber twist behavior. The maximum stress was chosen as the damage initiation criterion, in which damage was defined to initiate when the maximum nominal stress ratio reached a critical value. The stiffness started to degrade after the damage initiation. The Benzeggagh–Kenane (BK) damage evolution criterion was adopted.

(1) \begin{equation*} G_n^c + (G_s^c - G_n^c)\left\{ {\frac{{{G_S}}}{{{G_T}}}} \right\}^{\eta} = {G^c} \end{equation*}

where the material parameter $\eta = 1$ and $G_n$, $G_s$, $G_t$ represent the work done by the tractions and their conjugate relative displacements in the normal (*n*), first (*s*) and second (*t*) shear directions, respectively. $G_S$ is the portion of the total work done by the shear traction and the corresponding relative displacement components, and $G_S = G_s + G_t$. $G_T$ is the total work done by external forces, and $G_T = G_n + G_S$. The critical fracture energy $G_n^c = G_s^c = G_t^c = 15$ N⋅mm$^{-2}$. Once the energy release rate $G\left(G = G_n + G_t + G_s\right)$ exceeds the critical energy release rate $G_c$, the contact faces totally fracture.

## Supplementary Material

nwz077_Supplemental_Files
