# Supplementary material for: Bioinspired hierarchical helical nanocomposite macrofibers based on bacterial cellulose nanofibers
Source: Natl Sci Rev. 2019 Jun 21;7(1):73–83. doi: 10.1093/nsr/nwz077 (PMC8289019; doi:10.1093/nsr/nwz077)
Supplement: nwz077_Supplemental_Files [file nwz077_supplemental_files.zip › Supplementary data.docx]

**Bioinspired hierarchical helical nanocomposite macrofibers based on bacterial cellulose nanofibers**

Huai-Ling Gao1,🟉, Ran Zhao1,🟉, Chen Cui1, Yin-Bo Zhu2, Si-Ming Chen1, Zhao Pan1, Yu-Feng Meng1, Shao-Meng Wen1, Chuang Liu2, Heng-An Wu2 and Shu-Hong Yu1,2,*

1 Division of Nanomaterials & Chemistry, Hefei National Laboratory for Physical Sciences at the Microscale, CAS Center for Excellence in Nanoscience, Hefei Science Center of CAS, Department of Chemistry, University of Science and Technology of China, Hefei 230026, China.

2 CAS Key Laboratory of Mechanical Behavior and Design of Materials, Department of Modern Mechanics, CAS Center for Excellence in Complex System Mechanics, University of Science and Technology of China, Hefei 230027, China.

🟉 These authors contributed equally to this work.

* Correspondence and requests for materials should be addressed to the author: Shu-Hong Yu

Email: [shyu@ustc.edu.cn](mailto:shyu@ustc.edu.cn), Tel: 86-551-63603040, Fax: 86-551-63603040

**ABSTRACT**

Bio-sourced nanocellulosic materials are promising candidates for spinning high-performance sustainable macrofibers for advanced applications. Various strategies have been pursued to gain nanocellulose-based macrofibers with improved strength. However, nearly all of them have achieved at the expense of their elongation and toughness. Inspired by the widely existed hierarchical helical and nanocomposite structural features in biosynthesized fibers exhibiting exceptional combination of strength and toughness, we report a design strategy to make nanocellulose-based macrofibers with similar characteristics. By combining a facile wet-spinning process with a subsequent multiple wet-twisting procedure, we successfully obtain biomimetic hierarchical helical nanocomposite macrofibers based on bacterial cellulose nanofibers, realizing impressive improvement of their tensile strength, elongation and toughness simultaneously. The achievement certifies the validity of the bioinspired hierarchical helical and nanocomposite structural design proposed here. This bioinspired design strategy provides a potential platform for further optimizing or creating many more strong and tough nanocomposite fiber materials for diverse applications.

**Keywords:** bioinspired, nanocomposite, hierarchical helical macrofibers, strength and toughness, bacterial cellulose

**INTRODUCTION**

High-performance biomass-based nanocomposites are emerging as advanced renewable and sustainable materials for future structural and functional applications [1-9]. Bio-sourced nanocellulosic materials, the most abundant raw material systems on earth, have attracted tremendous scientific and commercial attention recently due to their attractive combination of many inherent merits in terms of biodegradability, low density, thermal stability, global availability from renewable resources, as well as impressive mechanical properties [10,11]. These features make them promising candidates for the development of mechanically robust, sustainable and biocompatible materials for diverse applications [12-14].

Nanofibrillated cellulose (NFC) [13] and cellulose nanocrystals (CNC) [15] obtained from plants and bacterial cellulose (BC) nanofibers obtained via bacterial fermentation [16] represent a remarkable class of nature-derived nanofibers with superior intrinsic mechanical properties owing to their high degree of polymerization and crystallinity. These extremely fine natural polymeric nanofibers have been intensively investigated for fabricating high-performance macrofibers. Various strategies, such as flow-assisted assembly [13], combining wet-spinning with mechanical stretching [17,18] or chemical crosslinking [14], mixing synergetic constituents together [19], have been pursued to strengthen the nanofiber alignment or enhance the interfibrillar interactions, *etc*. As a result, significant enhancements in strength and stiffness have been achieved of resultant nanocellulose-based macrofibers. However, as strength and toughness are always mutually exclusive for man-made structural materials [20], almost all the achievements ultimately came at the expense of elongation and toughness of the obtained macrofibers. For example, mechanical stretching can improve the CNF/CNC orientation resulting in marked improvement of tensile strength and stiffness, but meanwhile leads to obvious embrittlement and low failure strain [21,22]. Generally, compared with strength and stiffness, elongation and toughness are even more critical for fiber materials, especially for those relative to textile applications [23-25]. Therefore, this dilemma is quite common for previously reported nanocellulose-based macrofibers. Besides pursuing high tensile strength, further improving their elongation and toughness is still a significant challenge.

Nature can always provide inspirations for us to remedy this troublesome conflict between strength and toughness [26]. The widespread biosynthesized fibers, ranging from various lignocellulosic fibers in plants [11] to spider silk [27], collagen fibers and animal hairs [28], are all featured by exceptional combination of high tensile strength and toughness. This performance combination is mainly attributed to their hierarchical helical structures across multiple length scales with stiff and strong nanoscale fibrous building blocks embedded in softer and energy dissipating matrices [29]. These soft matrices are supposed to play critical roles in the mechanical behavior by providing favorable interfaces between the fibrous units. Typically, CNFs in the outer cell wall layer (S2 layer) of plants are aligned and embedded in a matrix of lignin and hemicellulose to form strong and tough nanocomposite helical fibers.

Herein, inspired by the above motivations, we propose a hierarchical helical and nanocomposite structural design strategy to fabricate mechanically robust macrofibers. The obtained macrofibers are expected to be composed of helically aligned BC nanofibers embedded in a soft biopolymer matrix (Fig. 1a). By combining a facile wet-spinning process with a subsequent multiple wet-twisting and drying procedure, the structural and mechanical features in natural biosynthesized fibers are supposed to be transferred into the designed artificial macrofibers, achieving hierarchical helical nanocomposite macrofibers with a simultaneous improvement of tensile strength, elongation and toughness, which is challenging for most previously reported nanocellulose-based macrofibers. The expected combination of desirable mechanical properties will endow the macrofibers with promising potential for further advanced applications.

**RESULTS AND DISCUSSION**

**Fabrication and structural characterization**

As an alternative to plant derived CFC and CNC, BC nanofibers are much purer, have larger aspect ratio and higher degree of crystallinity and polymerization [30-32], which make them a particularly appealing kind of building blocks for fabricating mechanical robust macrofibers. Here, dispersed BC nanofibers with diameter of approximately 60 nm and length of dozens of micron (Fig. 1b-d and Supplementary Fig. 1a) were utilized as the structural units to prepare our hierarchical helical nanocomposite macrofibers. Sodium Alginate (Alg), a biodegradable and biocompatible anionic polysaccharide with abundant carboxylic acid groups, was chosen as the soft matrix between BC nanofibers. The hydroxyl groups in BC [31] can interact with the carboxylic acid groups of Alg, forming strong hydrogen bonding (Supplementary Fig. 1b). Thus, good interfacial interaction between BC nanofibers and Alg can be achieved. This interfacial interaction is supposed to be beneficial for stress transfer between the two components, which is essential for good mechanical properties of the relevant composites [33].

For fabricating the hierarchical helical nanocomposite macrofibers, aqueous dispersions of BC-Alg mixture were first spun into continuous BC-Alg gel filaments consisting of uniaxial orientation of BC nanofibers embedded in the Alg matrix (Supplementary Fig. 1c). A facile wet-spinning method widely used in previous works to facilitate uniaxial orientation of other nanofibers under shear force from flows [34] was applied here. Unlike the classical wet-spinning method applied in industry, where high concentration polymer solutions are extruded through thousands of capillaries into the coagulation bathes to produce yarns of fibers at huge amounts, the wet-spinning process used here was simplified to demonstrate our study. To be specific, BC-Alg dispersions were extruded through a single capillary needle into coagulation bathes of CaCl2 aqueous solution to form continuous BC-Alg gel filaments (Fig. 1e and Supplementary Movie 1). During the extruding process, BC nanofibers were forced to align paralleled to the long axis of the needle under local shear force at the solid boundaries of the nozzle [17]. Then the orientation of BC nanofibers was fixed immediately upon Ca2+ induced coagulation of the Alg matrix (Supplementary Fig. 1d). In order to introduce hierarchical helical structure into ultimate macrofibers, the obtained BC-Alg gel filaments (Fig. 3f) were then twisted together according to a proposed multilevel wet-twisting process as illustrated in Fig. 1a. For simplicity, every two sub-level gel filaments were twisted together at each hierarchical level (Fig. 3g, Supplementary Fig. 2 and Supplementary Movie 1) to verify our design strategy. The twisted gel filaments with different hierarchical levels were then air dried to obtain the hierarchical helical nanocomposite macrofibers with dense structures.

Scanning electron microscope (SEM) observation revealed that both the albert lay (+) and interactive lay (-) helical BC-Alg macrofibers after drying presented slightly twisted alignment of surface textures (Fig. 2a-d and Supplementary Figs. 3-5). The interface between original separate filaments was ambiguous with only a few packing defects or voids, which should be disappeared during the drying process. Before drying, the gel filaments were actually hydrogels, containing large amounts of water. The calcium alginate macromolecular chains in the gel filaments were in a relatively relaxed state. The gel filaments had a good wettability due to their high water content. When several single gel filaments were twisted together, a thin water film on the surface would further bind the gel filaments closer to each other due to surface tension. In this state, the outermost polymer segments would penetrate into other surfaces as a result of their high local mobility [35]. Thus, during the drying process, the entanglement (interpenetration) of the surface segments was supposed to happen under the van der Waals force among them. Therefore, the original interfaces among the gel filaments disappeared slowly, and the original separate gel fibers integrated into a relatively dense single macrofibers with only a few voids. Note that the helical macrofibers exhibit good flexibility and knitting property (Fig. 2d insert), indicating their promising potential for textile applications. From a close-up of the surface of single BC-Alg filament shown in Fig. 2e, a crumpled texture with direction along its long axis was observed, which was attributed to the lateral contraction during the drying process, because both two ends of the macrofibers were fixed before drying. This lateral contraction should further contribute to the orientation of the embedded BC nanofibers. As we expected, BC nanofibers in the single BC-Alg filament were homogeneously distributed and almost paralleled oriented along its long axis (Fig. 2f, g and Supplementary Fig. 6a-c). High magnification cross-sectional SEM image shows that individual BC nanofibers have been pulled out from the Alg matrix on the fracture surface (Fig. 2g), indicating efficient stress transfer and great energy dissipation during rupture.

An identification of the orientation of BC nanofibers in the microfibers was further assessed via wide-angle X-ray diffraction (WAXD). In stark contrast to that of the BC-Alg film with randomly oriented BC nanofibers in the horizontal direction (Supplementary Fig. 6d), the (200) reflection used to quantify the orientation of cellulose crystals [13] was presented as an arc pattern in the diffractogram of the dried BC-Alg macrofibers and the azimuthal intensity profiles of the (200) reflection was prominent (Fig. 2h), suggesting the highly aligned orientation of BC nanofibers in the nanocomposite BC-Alg macrofibers [36]. Moreover, the high-orientation of BC nanofibers in the dried hierarchical helical macrofibers was then confirmed by the brilliant color under crosspolarized light due to birefringence phenomenon of BC nanofibers caused by their oriented structure [17]. As shown in Fig. 2i, j and Supplementary Figs. 7-9, both the Helical-4 and Helical-8 macrofibers with albert lay (+) and interactive lay (-) reveal obvious brilliant colors with slightly twisted directions. Note that each original BC-Alg filament with twist state in the macrofibers can be distinguished. These results can partly confirm the helical spatial distribution of BC nanofibers in the nanocomposite macrofibers over large scale dimension.

**Mechanical properties**

Systematical mechanical investigations were carried out to establish a quantitative correlation between the mechanical performance and the designed structures. It was found that tensile strength of the dried BC-Alg filaments showed a consistent increase with a rising content of BC nanofibers up to a weight ratio of 40 wt.% and then rapidly dropped (Fig. 3a and Supplementary Fig. 10a). The optimal filament showed more than double and nearly four times of the original tensile strength of the neat Alg filament and the disordered BC-Alg film with the same BC content, respectively (Supplementary Fig. 10). These results greatly certify the contribution of BC alignment to the ultimate mechanical properties, and also reveal that a proper amount of soft matrix is crucial for efficient stress transfer between BC nanofibers, leading to impressive mechanical properties of the ultimate nanocomposite filaments (Fig. 3b). The optimized twist level was further identified to be 100 twist per meter (TPM) for the Helical-2 BC-Alg macrofibers in consideration of the optimum combination of strength and elongation. As shown in Fig. 3b, both the strength and strain at break rise similarly with the rising twist levels before a critical point (2-100) and then begins to decrease. It can be hypothesized that increasing the twist level would cant the BC nanofibers and improve the inter-filament interaction under axial tension leading to an increase in the macrofiber strength before the optimum point, after which the nanofiber obliquity in the macrofiber becomes unsuitable leading to a drop in the macrofiber strength. This phenomenon matches the mechanical behavior found in traditional twisted yarns [37]. Besides, the prestressing force induced by wet-twisting and drying process might also correlate to the strength of the resultant macrofibers.

The effect of helical hierarchy on the mechanical properties of resultant macrofibers was further investigated. We found that the albert lay (-) BC-Alg macrofibers exhibited a little higher tensile strength and smaller elongation, respectively, than those of the interactive lay (+) BC-Alg macrofibers at each hierarchical level (Fig. 3c-e and Table 1). Note that the tensile strength continuously increased for the Helical-4 macrofibers, then dropped for the Helical-8 macrofibers, while both albert lay (+) and interactive lay (-) BC-Alg macrofibers showed continuous increase of strain at break as the hierarchical levels increased (Fig. 3c-e and Table 1). Consequently, impressive enhancement of toughness compared with those of the single BC-Alg filament and the disordered BC-Alg film was achieved, respectively (Fig. 3f and Table 1). In sharp contrast, all the macrofibers made from same number of gel filaments without twisting show much smaller tensile strength, strain at break (nearly no change) and toughness than those of the helical macrofibers (Fig. 3d-f and Table 1). It is worth highlighting that in most traditional nanocomposites, higher strength is typically achieved at the expense of strain at break and toughness. However, we achieved a simultaneous increase of strength, elongation and toughness in our bioinspired macrofibers. This mechanical enhancement effect was also achievable for the bioinspired macrofibers conditioned at different relative humidity (Supplementary Fig. 11 and Supplementary Table 1). These experimental findings provide direct evidences for the positive contribution of our bioinspired hierarchical helical and nanocomposite structural design to the mechanical properties of resultant macrofibers. The strength decreases of the Helical-8 and untwisted macrofibers are supposed to be mainly caused by some inevitable packing voids or defects derived during their drying processes. In these processes, the wet gel filaments changed into dried macrofibers with a large radial shrinkage (about 10 times) due to the loss of plenty of water. Thus, some packing voids or defects among the gel filaments are considered to be inevitable during this process, which would have a negative impact on the mechanical strength of both the helical fibers and the untwisted macrofibers. The increases of tensile strength of the Helical-2 and Helical-4 fibers should be attribute to the relative larger contribution of the bioinspired structural design, and this negative effect became dominant with the increase of initial gel filament numbers.

Owing to the helical structure, both the albert lay (+) and interactive lay (-) BC-Alg macrofibers would rotate under tensile loading, and the albert lay (+) case is especially prone to this tendency according to the traditional theory for rope and rope-like materials [38]. During this rotation process, each helically distributed filaments in the macrofibers is gradually straightened before break, leading to elongation increase of the whole helical BC-Alg fibers. It is assumed that helical fibers with higher hierarchical levels exhibit larger elongation potential due to the multi-level rotational deformations. Nonlinear finite element simulations further revealed a good consistency with this speculation. In sharp contrast to the untwisted fiber model, both the Helical-2 and Helical-4 fiber models revealed obvious rotation phenomenon under uniaxial tension (Fig. 4, Supplementary Fig. 10 and Supplementary Movie 2), resulting in conspicuous improvement of tensile deformation before the fibers break relative to the untwisted one (Supplementary Fig. 10c). Thus, we can infer why the elongation of the interactive lay (-) BC-Alg macrofibers was found to be a little smaller relative to the albert lay (+) BC-Alg macrofibers and much larger than that of the untwisted macrofibers. Moreover, during the uniaxial tension, multiscale deformation with interfacial frictional sliding of the discontinued BC nanofibers in Alg matrix at each helical hierarchy would happen synchronously, giving rise to much energy dissipation [25,39,40]. Consequently, attractive improvements of toughness for both the albert lay (+) and interactive lay (-) macrofibers were achieved (Fig. 3e, f).

It was obvious that the obtained hierarchical helical macrofibers exhibit a distinguishing mechanical improvement with a simultaneous increase of strength, elongation and toughness relative to the untwisted macrofibers, providing a valuable solution for the traditional dilemma of most man-made fiber materials. It can be found that in most previously reported nanocellulose-based macrofibers, there tends to be a regular phenomenon that increasing the tensile strength is always achieved at the expense of elongation and toughness. In contrast, the elongation and toughness increased by more than ~50% and 100%, respectively, for the Helical-4 (-) macrofibers with simultaneous increase of tensile strength (Fig. 5a). The unique property combination of our designed macrofibers should be attributed to the synergistic effects of the bioinspired hierarchical helical and nanocomposite structure. As shown in Fig. 5b, c and Supplementary Table 2, though the achieved maximum tensile strength (~535 MPa) is still lower than those of some mature industrial regenerated cellulose fibers (Lyocell, Cordenka and Ioncell-F fibers) and several nanocellulose-based spun fibers [14,19,53], it is comparable to those of high-quality biosynthesized fibers and outperforms those of most nanocellulose-based macrofibers in previous works. Furthermore, the elongation (with maximum average value of ~16%) surpasses nearly all those of the biosynthesized and the reported man-made nanocellulose-based fibers. As a result, the bioinspired hierarchical helical macrofibers display impressive mechanical superiority, especially when considering both the toughness (with maximum value of ~45 MJ m-3) and elongation (Fig. 5d and Supplementary Table 2), certifying the validity of the bioinspired hierarchical helical and nanocomposite structural design proposed here. Additionally, it should be noted that though the designed bioinspired hierarchical helical macrofibers share some similarity in terms of helical hierarchy and performance characteristics with traditional rope materials [38], benefits derived from the intrinsic merits of nanoscale building blocks and the superior nanocomposite structure are prominent.

**CONCLUSION**

In this study, with regard to the general problem of low elongtation or brittleness of previously reported nanocellulose-based macrofibers, we reported a bioinspired hierarchical helical and nanocomposite structural design strategy to fabricate mechanically robust macrofibers via combining a facile wet-spinning process with a subsequent multiple wet-twisting together. The resultant macrofibers exhibit a hierarchical helical structure with good alignment of BC nanofibers embedded in a soft Alg matrix in the macrofibers. This structural feature is recognized to give rise to the distinguishing mechanical improvement of the resultant nanocomposite macrofibers. The bioinspired structural design strategy presented here is simple, mild and valid, representing a promising platform for the development of high-performance nanocomposite fiber materials for future structural or functional applications, such as advanced textiles.

**METHODS**

**Fabrication of the hierarchical helical BC-Alg macrofibers**

Dispersed BC nanofibers were purchased from Qihong Technology Co., Ltd. (Guilin, Guangxi, China). SA were purchased from Aladdin Chemical Reagent Co. and used without further purification. Calcium chloride (CaCl2) was purchased from Sinopharm Chemical Reagent Co. Alg solution (20 mg ml-1) was mixed with BC nanofibers dispersions (4.5 mg ml-1) together by intensely stirring for ~60 min at room temperature to prepare the BC-Alg dispersions with certain content of BC (0 to 50 wt.%), followed by vacuum-pumping treatment to remove air bubbles. The resulting BC-Alg dispersions were then loaded into a plastic syringe and extruded through a capillary needle (a steel tube with an inner diameter of 0.41 mm and a length of 15 cm) into a CaCl2 coagulation bath (0.1 M) to form continuous BC-Alg gel filaments. The injection was controlled by an air pump operating at a pressure of ~20 psi. The continuous spun gel filaments were rolled onto a drum (Fig. 1e, f and Supplementary Movie 1) after soaking in the coagulation bath for 10 min. The collected gel filaments were then immersing in deionized water (DIW) to remove excess Ca2+. Afterwards, two single BC-Alg gel filaments were hung on a rotator of homemade twisting machine with two ends fixed without any drawing (Supplementary Fig. 2), and then twisted together into hierarchical helical BC-Alg mcarofibers with certain twist level according a multilevel wet-twisting process (Fig. 1a,g and Supplementary Movie 1). The optimized twist level for Helcial-2, 4 and 8 macrofibers are 100, 67 and 45 TPM, respectively, to achieve similar twist angle for them. At each hierarchical level, every two sub-level filaments were twisted together. The rotate directions were changed to obtain Helical (+)/(-) fibers. The obtained gel macrofibers were finally hung on a shelf at room temperature with a relative humidity of 50% for air drying. In this process, we first fixed one end of the gel macrofibers on a shelf and let the fibers suspend by gravity, then we fixed the other end without any drawing. The untwisted macrofibers consisting of different numbers of single filaments were prepared by binding these single filaments together paralleled and drying under the same condition as that of the hierarchical macrofibers. The disordered BC-Alg film was prepared by self-evaporation of BC-Alg dispersions containing 40 wt.% BC nanofibers. The dried film was then immersed with CaCl2 solution (0.1 M) for one hour followed by washing and air drying.

**Structure characterizations**

Transmission electron microscope (TEM) with a Hitachi H-7650 apparatus at an acceleration voltage of 120 kV was used to observe the BC nanofibers. The microstructure of BC nanofibers, the obtained BC-Alg macrofibers and films were observed by scanning electron microscope (SEM) (Zeiss Supra 40) at an acceleration voltage of 5 kV. Atomic force microscope (AFM) measurement was carried out on a Veeco DI Nanoscope MultiMode V system in the tapping mode. X-Ray Diffraction (XRD) patterns were achieved on a PW1710 instrument with CuKα radiation (*λ*=0.15406 nm). Fourier Transform Infrared Spectroscopy (FTIR) spectra were obtained from a Bruker Vector-22 FTIR spectrometer at room temperature.

**Orientation characterizations**

Optical microscope image between crossed polarizers was obtained with a polarizing microscope (Leica DM2700P, Germany) equipped with a Leica MC190 HD camera. Two-dimension (2D) WAXS measurements were carried out to monitor the evolution of structures. The X-ray wavelength was 0.154 nm and a Mar345 CCD detector (150 × 150 pixels) was employed to collect time-resolved 2D WAXD patterns. A bundle of BC-Alg macrofibers were placed in a sample holder perpendicular to the X-ray beam. The distance between the detector (Mar 345) and the sample was 195.00 mm. A typical acquisition time was 60 s. The patterns were corrected for air scattering and background. Fit2D software from the European Synchrotron Radiation Facility was used to analyze the data.

**Mechanical testing**

For samples tested at different relative humidity (RH), the samples were first conditioned at 45% RH, 50% RH and 65% RH, respectively, for at least 24 h prior to testing. Then the samples were tested immediately in uniaxial tension at room temperature using Instron 5565 A equipped with a 10 N and 500 N load cells. For the macrofibers dried in completely dry environment, they were conditioned in an 80 °C oven for 24h first, and then roasted using an infrared lamp beside the samples when they were under mechanical testing to avoid absorbing moisture from the air. At least 6 specimens were tested for all the values presented. The specimens were cutting into ~30 mm long. Tests were performed at a loading rate of 1 mm min-1 with a gauge length of ~10 mm. The tensile strength was calculated by using the fiber cross-sectional areas from optical microscope images (cross-checked with SEM images) which were measured by IMAGE J.

**Finite element analysis**

Three models with different structures consisting of two parallel cylinders, two spiral cylinders and two secondary spiral cylinders, respectively, were built as shown in Fig. 3g-i and Supplementary Fig. 12a. Three-dimensional nonlinear finite element simulations were performed using the commercial software ABAQUS. In the simulation, cohesive zone models were used at the half of the fiber structure to model the failure of our designed material structures, where a bi-linear traction-separation (TS) law was adopted, as shown in Supplementary Fig. 12b. The fibers with isotropic elastic modulus *E* = 14.5 GPa, Poison ratio *ν* = 0.31 suffered from elastic and plastic deformation before failure. The parameters *Knn*, *Kss*, and *Ktt* were set to be 14.5 GPa. A mixed mode fracture was taken into consideration due to the fiber twist behavior. The max stress was chosen as the damage initiation criterion, in which damage was defined to initiate when the maximum nominal stress ratio reaches a critical value. The stiffness started to degrade after the damage initiation. Benzeggagh and Kenane (BK) damage evolution criterion was adopted.

(1)

where The critical fracture energy 15 N·mm-2. Once the energy release rate exceed the critical energy release rate , the contact faces totally fracture.

**SUPPLEMENTARY DATA**

Supplementary data are available at NSR online.

**ACKNOWLEDGEMENTS**

We thank Liang-Bin Li, Qiang Ding and Zhong-Liang Zhu for assistance. This work was partially carried out at the USTC Center for Micro and Nanoscale Research and Fabrication.

**FUNDING**

This work was supported by the National Natural Science Foundation of China (Grants 51732011, 51702310, 21431006, 21761132008, 11525211, 11872063, 11802302), the Foundation for Innovative Research Groups of the National Natural Science Foundation of China (Grant 21521001), Key Research Program of Frontier Sciences, CAS (Grant QYZDJ-SSW-SLH036), the National Basic Research Program of China (Grant 2014CB931800), the Strategic Priority Research Program of the Chinese Academy of Sciences (XDB22040402), the Users with Excellence and Scientific Research Grant of Hefei Science Center of CAS (2015HSC-UE007), the Anhui Provincial Natural Science Foundation (1808085ME115), the National Postdoctoral Program for Innovative Talents (BX201700225), the Fundamental Research Funds for the Central Universities (WK2060190076, WK2090050040, WK6030000067).

**AUTHOR CONTRIBUTIONS**

S.-H.Y. and H.-L.G. conceived the idea and designed the experiments. S.-H.Y. supervised the research. H.-L.G. and R.Z. performed the experiments and analyzed the data. C.C., Y.-B.Z. and H.-A.W. performed theoretical analyses. S.-M.C., Z.P., Y.-F.M. and S.-M.W. helped to perform some experiments and analyze the data. H.-L.G., R.Z., C.C. and S.-H.Y. co-wrote the manuscript. All authors discussed the results.

*Conﬂict of interest statement.*None declared.

**REFERENCES**

1 Zhu H, Luo W and Ciesielski PN *et al.* Wood-Derived Materials for Green Electronics, Biological Devices, and Energy Applications. *Chem Rev* 2016; **116:** 9305-9374.

2 Song J, Chen C and Zhu S *et al.* Processing bulk natural wood into a high-performance structural material. *Nature* 2018; **554:** 224-228.

3 Ling SJ, Qin Z and Huang WW *et al.* Design and function of biomimetic multilayer water purification membranes. *Sci Adv* 2017; **3:** e1601939.

4 Ling S, Jin K and Qin Z *et al.* Combining In Silico Design and Biomimetic Assembly: A New Approach for Developing High-Performance Dynamic Responsive Bio-Nanomaterials. *Adv Mater* 2018; **30:** 1802306.

5 Gao HL, Chen SM and Mao LB *et al.* Mass production of bulk artificial nacre with excellent mechanical properties. *Nat Commun* 2017; **8:** 287.

6 Mao LB, Gao HL and Yao HB *et al.* Synthetic nacre by predesigned matrix-directed mineralization. *Science* 2016; **354:** 107-110.

7 Si Y, Wang X and Yan C *et al.* Ultralight Biomass-Derived Carbonaceous Nanofibrous Aerogels with Superelasticity and High Pressure-Sensitivity. *Adv Mater* 2016; **28:** 9512-9518.

8 Bai H, Chen Y and Delattre B *et al*. Bioinspired large-scale aligned porous materials assembled with dual temperature gradients. *Sci Adv* 2015; **1:** e1500849.

9 Wicklein B, Kocjan A and Salazar-Alvarez G *et al.* Thermally insulating and fire-retardant lightweight anisotropic foams based on nanocellulose and graphene oxide. *Nat Nanotechnol* 2015; **10:** 277-283.

10 Ling S, Kaplan DL and Buehler MJ. Nanofibrils in nature and materials engineering. *Nat Rev Mater* 2018; **3:** 18016.

11 Klemm D, Heublein B and Fink HP *et al*. Cellulose: Fascinating biopolymer and sustainable raw material. *Angew Chem Int Edit* 2005; **44:** 3358-3393.

12 Kontturi E, Laaksonen P and Linder MB *et al.* Advanced Materials through Assembly of Nanocelluloses. *Adv Mater* 2018; **30:** e1703779.

13 Håkansson KMO, Fall AB and Lundell F *et al.* Hydrodynamic alignment and assembly of nanofibrils resulting in strong cellulose filaments. *Nat Commun* 2014; **5:** 4018.

14 Mittal N, Ansari F and Gowda.V K *et al.* Multiscale Control of Nanocellulose Assembly: Transferring Remarkable Nanoscale Fibril Mechanics to Macroscale Fibers. *ACS Nano* 2018; **12**: 6378-6388.

15 Wang BC and Walther A. Self-Assembled, Iridescent, Crustacean-Mimetic Nanocomposites with Tailored Periodicity and Layered Cuticular Structure. *ACS Nano* 2015; **9:** 10637-10646.

16 Gatenholm P and Klemm D. Bacterial Nanocellulose as a Renewable Material for Biomedical Applications. *MRS Bulletin* 2011; **35:** 208-213.

17 Yao J, Chen S and Chen Y *et al.* Macrofibers with High Mechanical Performance Based on Aligned Bacterial Cellulose Nanofibers. *ACS Appl Mater Interfaces* 2017; **9:** 20330-20339.

18 Wang B, Torres-Rendon JG and Yu J *et al.* Aligned bioinspired cellulose nanocrystal-based nanocomposites with synergetic mechanical properties and improved hygromechanical performance. *ACS Appl Mater Interfaces* 2015; **7:** 4595-4607.

19 Mittal N, Jansson R and Widhe M *et al.* Ultrastrong and Bioactive Nanostructured Bio-Based Composites. *ACS Nano* 2017; **11:** 5148-5159.

20 Ritchie RO. The conflicts between strength and toughness. *Nat Mater* 2011; **10:** 817-822.

21 He Y, Zhang N and Gong Q *et al.* Alginate/graphene oxide fibers with enhanced mechanical strength prepared by wet spinning. *Carbohyd Polym* 2012; **88:** 1100-1108.

22 Kafy A, Kim HC and Zhai L *et al.* Cellulose long fibers fabricated from cellulose nanofibers and its strong and tough characteristics. *Sci Rep* 2017; **7:** 17683.

23 Ling S, Qin Z and Li C *et al.* Polymorphic regenerated silk fibers assembled through bioinspired spinning. *Nat Commun* 2017; **8:** 1387.

24 Ma T, Gao HL and Cong HP *et al.* A Bioinspired Interface Design for Improving the Strength and Electrical Conductivity of Graphene-Based Fibers. *Adv Mater* 2018; **30:** 1706435.

25 Zhang J, Feng W and Zhang H *et al.* Multiscale deformations lead to high toughness and circularly polarized emission in helical nacre-like fibres. *Nat Commun* 2016; **7:** 10701.

26 Wegst UG, Bai H and Saiz E *et al.* Bioinspired structural materials. *Nat Mater* 2015; **14:** 23-36.

27 Keten S, Xu Z and Ihle B *et al.* Nanoconfinement controls stiffness, strength and mechanical toughness of beta-sheet crystals in silk. *Nat Mater* 2010; **9:** 359-367.

28 Meyers MA, Chen P-Y and Lin AY-M *et al.* Biological materials: Structure and mechanical properties. *Prog Mater Sci* 2008; **53:** 1-206.

29 Fratzl P, Weinkamer R. Nature’s hierarchical materials. *Prog Mater Sci* 2007; **52:** 1263-1334.

30 Greca LG, Lehtonen J and Tardy BL *et al.* Biofabrication of multifunctional nanocellulosic 3D structures: a facile and customizable route. *Mater Horiz* 2018; **5:** 408-415.

31 Wu ZY, Li C and Liang HW *et al.* Ultralight, Flexible, and Fire-Resistant Carbon Nanofiber Aerogels from Bacterial Cellulose. *Angew Chem Int Edit* 2013; **52:** 2925-2929.

32 Czaja W, Romanovicz D and Brown RM. Structural investigations of microbial cellulose produced in stationary and agitated culture. *Cellulose* 2004; **11:** 403-411.

33 Jalal Uddin A, Araki J and Gotoh Y. Toward "strong" green nanocomposites: polyvinyl alcohol reinforced with extremely oriented cellulose whiskers. *Biomacromolecules* 2011; **12:** 617-624.

34 Clemons C. Nanocellulose in Spun Continuous Fibers: A Review and Future Outlook. *J Renew Mater* 2016; **4:** 327-339.

35 Maeda N, Chen NH and Tirrell M *et al*. Adhesion and friction mechanisms of polymer-on-polymer surfaces. *Science* 2002, **297:** 379-382.

36 Ureña-Benavides EE and Kitchens CL. Wide-Angle X-ray Diffraction of Cellulose Nanocrystal−Alginate Nanocomposite Fibers. *Macromolecules* 2011; **44:** 3478-3484.

37 Shah DU, Schubel PJ and Clifford MJ. Modelling the effect of yarn twist on the tensile strength of unidirectional plant fibre yarn composites. *J Compos Mater* 2013; **47:** 425-436.

38 Evans JJ and Ridge IML. Rope and rope-like structures. *WIT Transactions on State-of-the-art in Science and Engineering* 2005; **20:** 133-169.

39 Jia C, Chen C and Kuang Y *et al.* From Wood to Textiles: Top-Down Assembly of Aligned Cellulose Nanofibers. *Adv Mater* 2018; **30:** e1801347.

40 Das P, Heuser T and Wolf A *et al.* Tough and catalytically active hybrid biofibers wet-spun from nanochitin hydrogels. *Biomacromolecules* 2012; **13:** 4205-4212.

41 Toivonen MS, Kurki-Suonio S and Wagermaier W *et al.* Interfacial Polyelectrolyte Complex Spinning of Cellulose Nanofibrils for Advanced Bicomponent Fibers. *Biomacromolecules* 2017; **18:** 1293-1301.

42 Lee TW, Han M and Lee SE *et al.* Electrically conductive and strong cellulose-based composite fibers reinforced with multiwalled carbon nanotube containing multiple hydrogen bonding moiety. *Compos Sci Technol* 2016; **123:** 57-64.

43 Mohammadi P, Toivonen MS and Ikkala O *et al.* Aligning cellulose nanofibril dispersions for tougher fibers. *Sci Rep* 2017; **7:** 11860.

44 Torres-Rendon JG, Schacher FH and Ifuku S *et al.* Mechanical performance of macrofibers of cellulose and chitin nanofibrils aligned by wet-stretching: a critical comparison. *Biomacromolecules* 2014; **15:** 2709-2717.

45 Hooshmand S, Aitomaki Y and Norberg N *et al.* Dry-Spun Single-Filament Fibers Comprising Solely Cellulose Nanofibers from Bioresidue. *ACS Appl Mater Interfaces* 2015; **7:** 13022-13028.

46 Bledzki AK and Gassan J. Composites reinforced with cellulose based fibres. *Prog Polym Sci* 1999; **24:** 221-274.

47 Wambua P, Ivens J and Verpoest I. Natural fibres: can they replace glass in fibre reinforced plastics? *Compos Sci Technol* 2003; **63:** 1259-1264.

48 Chen XY, Guo QP and Mi YL. Bamboo fiber-reinforced polypropylene composites: A study of the mechanical properties. *J Appl Polym Sci* 1998; **69:** 1891-1899.

49 Walther A, Timonen JV and Diez I *et al.* Multifunctional high-performance biofibers based on wet-extrusion of renewable native cellulose nanofibrils. *Adv Mater* 2011; **23:** 2924-2928.

50 Lundahl MJ, Cunha AG and Rojo E *et al.* Strength and Water Interactions of Cellulose I Filaments Wet-Spun from Cellulose Nanofibril Hydrogels. *Sci Rep* 2016; **6:** 30695.

51 Nechyporchuk O, Bordes R and Kohnke T. Wet Spinning of Flame-Retardant Cellulosic Fibers Supported by Interfacial Complexation of Cellulose Nanofibrils with Silica Nanoparticles. *ACS Appl Mater Interfaces* 2017; **9:** 39069-39077.

52 Iwamoto S, Isogai A and Iwata T. Structure and mechanical properties of wet-spun fibers made from natural cellulose nanofibers. *Biomacromolecules* 2011; **12:** 831-836.

53 Wang S, Jiang F and Xu X *et al.* Super-Strong, Super-Stiff Macrofibers with Aligned, Long Bacterial Cellulose Nanofibers. *Adv Mater* 2017; **29:** 1702498.

**Figure Captions**

**
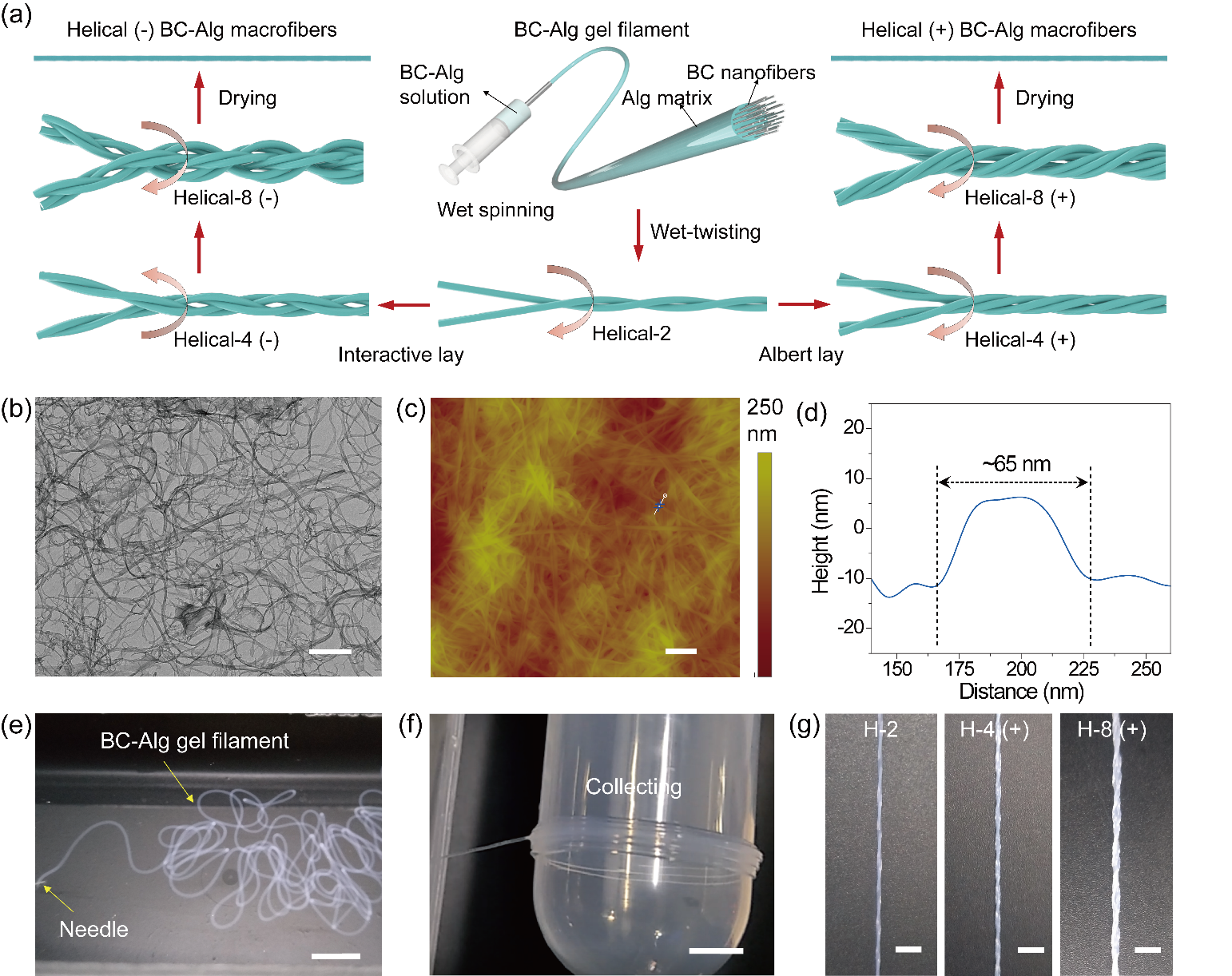
**

**Figure 1. Fabraction process of the hierarchical helical BC-Alg macrofibers.** (**a**) Schematic illustration of the fabrication process of the bioinspired hierarchical helical BC-Alg macrofibers. At each hierarchical level, every two sub-level gel filaments are twisted together to prepare a higher level helical fiber. Helical-2, 4 and 8 indicate the helical fibers composed of 2, 4 and 8 original filaments, respectively. The helical fibers with the same twist direction (albert lay) at each level are defined as Helical (+). The helical fibers with opposite twist direction (interactive lay) at each level are defined as Helical (-). (**b**) Transmission electron microscope (TEM) image of the dispersed BC nanofibers. Scale bar, 500 nm. (**c, d**) Atomic force microscope (AFM) image and its corresponding height profile (the dotted line) of the dispersed BC nanofibers. The measured diameter of a typical BC nanofiber is about 65 nm. Scale bar in (**c**), 500 nm. (**e**) Photograph shows that a continuous BC-Alg gel filament is extruded through a capillary needle into the coagulation bath of CaCl2 aqueous solution. Scale bar, 10 mm. (**f**) Photograph shows that a continuous BC-Alg gel filament is collected by a winding roller. Scale bar, 10 mm. (**g**) Photographs show the twisted state of a bundle of BC-Alg gel filaments after wet-twisting processes at each hierarchical level. Scale bars, 5 mm.

**
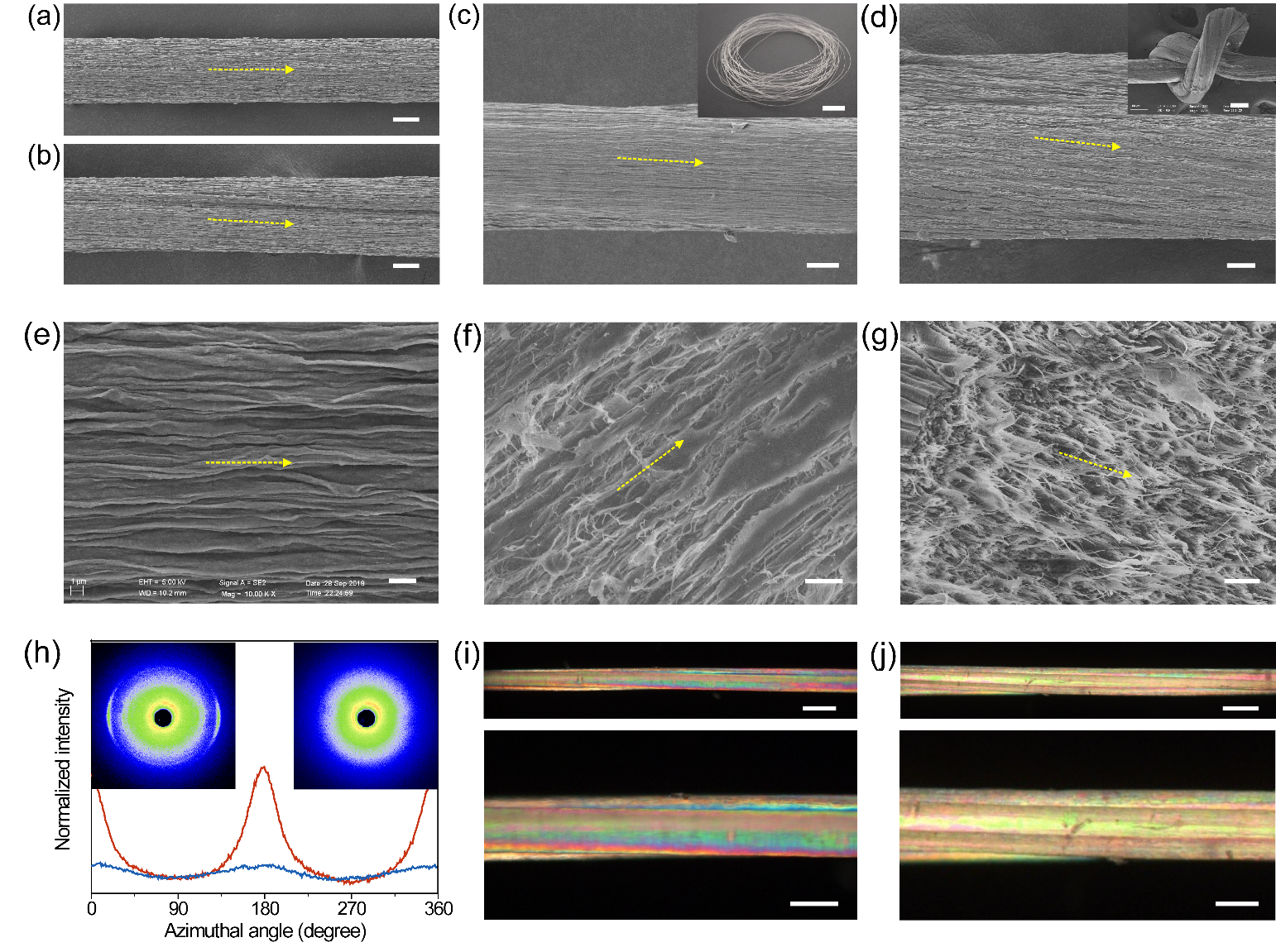
**

**Figure 2.** **Structural characterization of the hierarchical helical BC-Alg macrofibers.** (**a-d**)Lateral surface SEM images of single (**a**), Helical-2 (**b**), Helical-4 (+) (**c**) and Helical-8 (+) (**d**) BC-Alg macrofibers, where a slightly twisted surface texture (indicated by the yellow arrows) can be seen for thehelical BC-Alg fibers. Scale bars, 20 μm. The insert photograph in (**c**) is a roll of Helical-4 (+) BC-Alg macrofiber. Scale bar, 5 mm. The insert SEM image in (**d**) shows a knotted Helical-8 (+) BC-Alg macrofiber. Scale bar, 100 μm. (**e-g**) High-magnifcation lateral surface (**e**), longitudinal sectional (**f**) and cross sectional SEM images (**g**) of a typical single BC-Alg filament, showing the parallelly oriented BC nanofibers (indicated by the yellow arrows) along the long axis of the filament. Scale bars, 2 μm. (**h**) Azimuthal intensity profiles of the (200) scattering plane of the wide angle X-ray scattering diffracrograms of a bundle of helical BC-Alg macrofibers (right insert) and a BC-Alg film with randomly oriented BC nanofibers. (**i, j**)Optical micrographs with different magnifications of a Helical-4 (+) macrofiber (**i**) and a Helical-8 (+) macrofiber (**j**) between crossed polarizers, which both reveal typical birefringence with slightly twisted morphology. Scale bars, 200 μm for the upper and 100 μm for the lower micrographs, respectively.


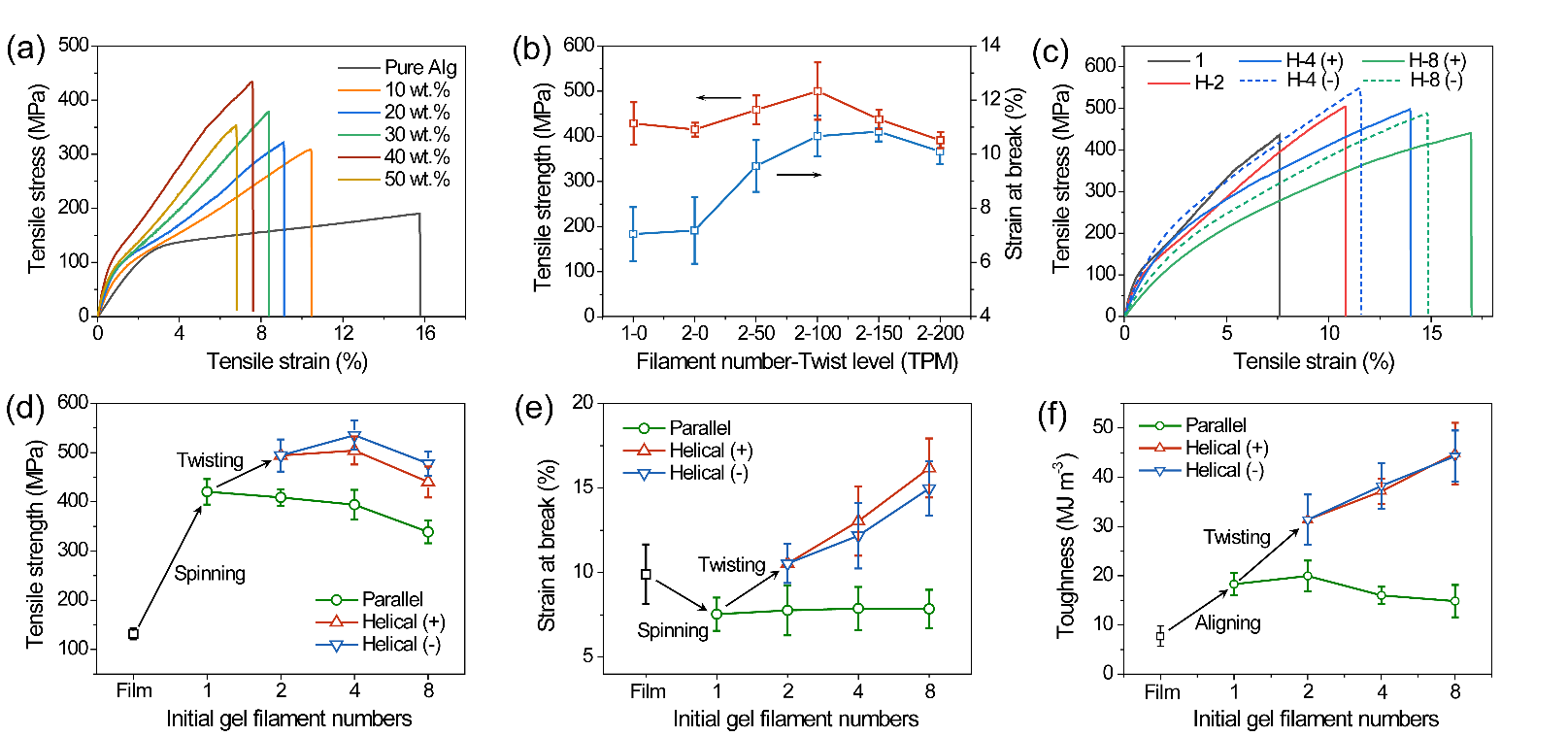


**Figure 3. Mechanical properties of the hierarchical helical BC-Alg mcarofibers.** (**a**)The typical stress-strain curves of the dried single BC-Alg filaments composed of different content of BC nanofibers. (**b**) The tensile strength as a function of twist level of the Helical-2 fibers. (**c**) The typical stress-strain curves of the hierarchical helical BC-Alg macrofibers with different hierarchical structures. (**d-f**)The tensile strength (**d**), strain at break (**e**) and toughness (**f**) as functions of hierarchical levels of the BC-Alg macrofibers. All the error bars represent the s.d. of at least six replicate measurements.


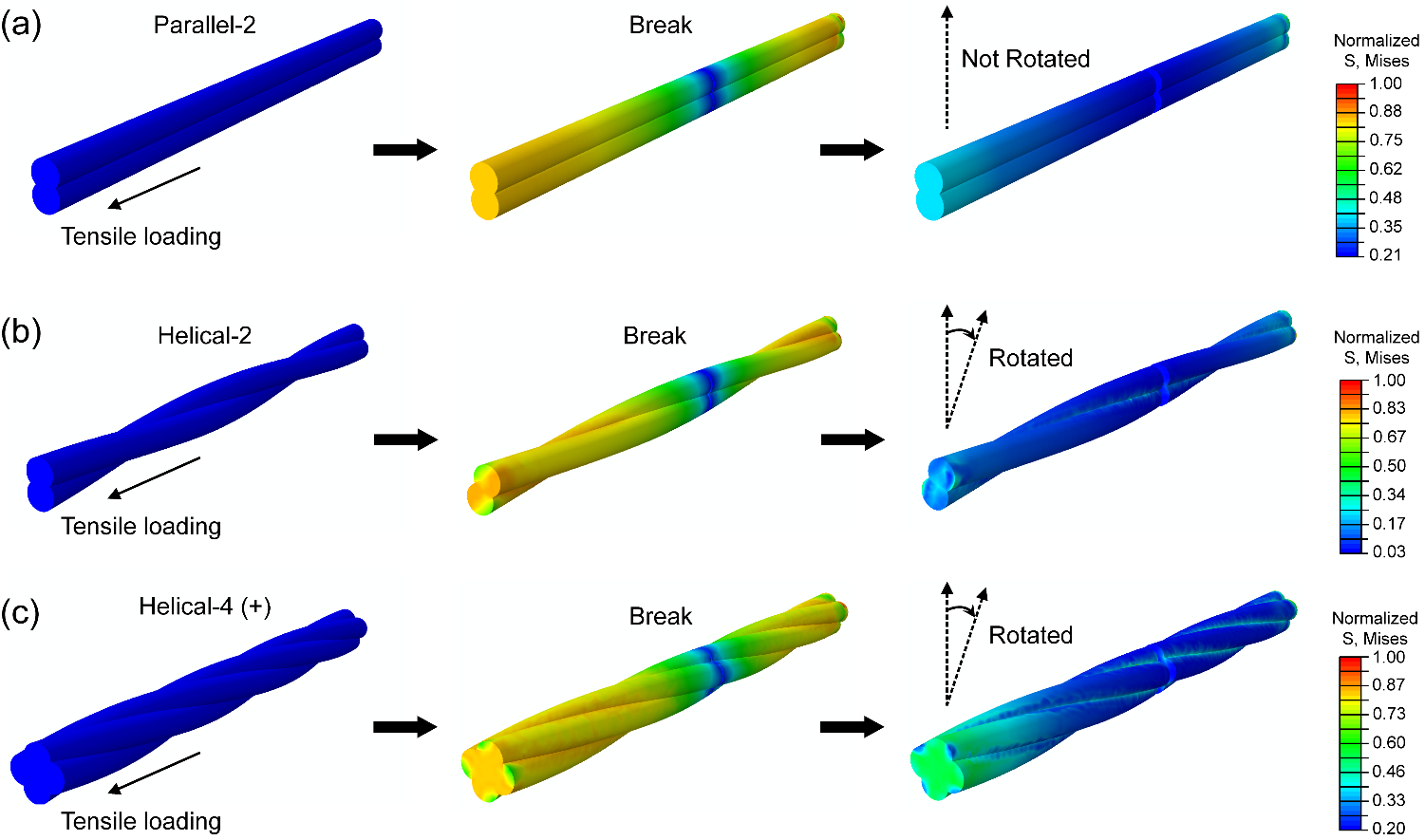


**Figure 4. Mechanical simulations of the fiber models with different structural features under tension loading.** (**a-c**)Nonlinear finite element simulations display three kind of fiber models under uniaxial tension processes. Note that both the Helical-2 and Helical-4 (+) fiber models would rotate in the tension processes before break. *θ* indicates the rotation degree of the simulated helical fibers from initial tension to rupture.


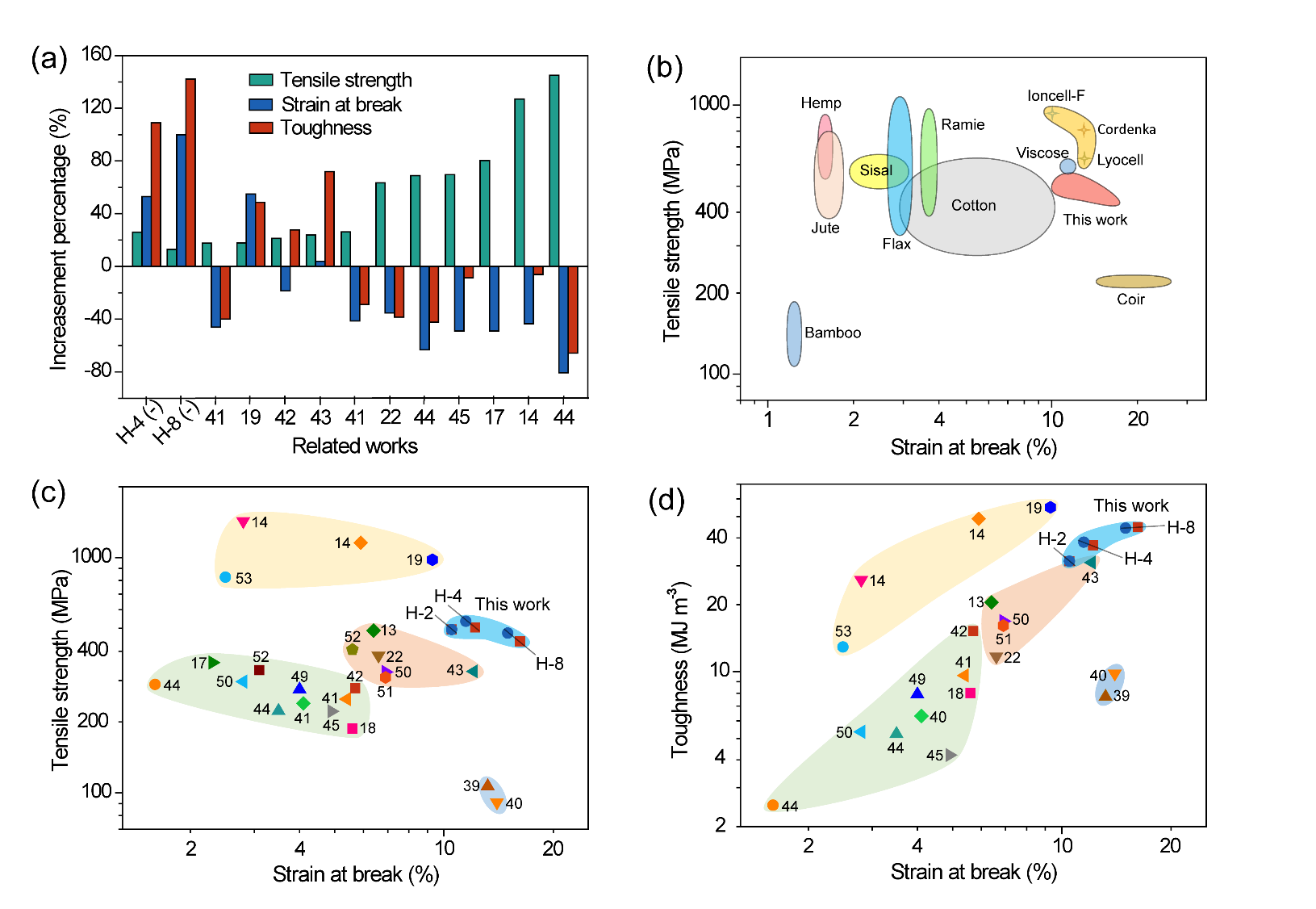


**Figure 5.** **Comparison of mechanical properties of our designed hierarchical helical macrofibers with their counterparts.** (**a**)Comparison of mechanical enhancement of the tensile strength, strain at break and toughness of our hierarchical helical macrofibers and previously reported cellulose based macrofibers [14,17,19,22,41-45]. (**b**) Ashby diagram of the tensile strength vs. strain at break of our hierarchical helical fibers compared with several mature industrial regenerated cellulose fibers and a wide range of biosynthetic cellulose based fibers [46-48]. (**c, d**) Ashby diagram of specific strength vs. strain at break (**c**) and toughness vs. strain at break (**d**) of our hierarchical helical fibers and previously reported cellulose based fibers [13,14,17-19,22,39-45,49-53]. Numbers in the charts stand for relevant references. Data plotted in the charts (**c, d**) was extracted from the maximum values given or estimated from stress-strain curves in these references. The increasement percentages of the tensile strength, strain at break and toughness in (**a**) were calculated by using the difference between the given values of the fibers without and with relevant design strategies.

**Table 1. Comparation of mechanical properties of the fabricated BC-Alg macrofibers with different structural features.** All the values were the mean value of at least six replicate measurements.

| **Initial gel filament numbers** |  | 1 | 2 | 4 | 8 |
| --- | --- | --- | --- | --- | --- |
| **Strength (MPa)** | Helical (+) | 420.4 | 494.0 | 504.2 | 440.3 |
|  | Helical (-) |  |  | 535.4 | 477.6 |
|  | Parallel |  | 408.9 | 394.2 | 338.7 |
| **Strain (%)** | Helical (+) | 7.5 | 10.5 | 13.0 | 16.2 |
|  | Helical (-) |  |  | 12.2 | 15.0 |
|  | Parallel |  | 7.8 | 7.9 | 7.9 |
| **Toughness (MJ m-3)** | Helical (+) | 18.3 | 31.4 | 37.1 | 44.8 |
|  | Helical (-) |  |  | 38.3 | 44.3 |
|  | Parallel |  | 20.0 | 16.0 | 14.8 |
